# High-power THz to IR emission by femtosecond laser irradiation of random 2D metallic nanostructures

**DOI:** 10.1038/srep12536

**Published:** 2015-07-24

**Authors:** Liangliang Zhang, Kaijun Mu, Yunsong Zhou, Hai Wang, Cunlin Zhang, X.-C. Zhang

**Affiliations:** 1 Department of Physics, Capital Normal University, No.105 XiSanHuan BeiLu, Beijing 100048, China; 2 The Institute of Optics, University of Rochester, Rochester, New York 14627, USA

## Abstract

Terahertz (THz) spectroscopic sensing and imaging has identified its potentials in a number of areas such as standoff security screening at portals, explosive detection at battle fields, bio-medical research, and so on. With these needs, the development of an intense and broadband THz source has been a focus of THz research. In this work, we report an intense (~10 mW) and ultra-broadband (~150 THz) THz to infrared (IR) source with a Gaussian wavefront, emitted from nano-pore-structured metallic thin films with femtosecond laser pulse excitation. The underlying mechanism has been proposed as thermal radiation. In addition, an intense coherent THz signal was generated through the optical rectification process simultaneously with the strong thermal signal. This unique feature opens up new avenues in biomedical research.

There has been considerable interest in the potential applications of radiation from the THz to IR range, which are also known as sub-millimeter waves, because these waves can penetrate many materials to reveal the corresponding spectral signatures. THz light is currently used in materials science research, security, pharmaceutical compound analysis, biomedical imaging, superconducting materials research, astronomy, and particle physics research^1^,^2^,^3^. However, this technology is used in few commercialized products because THz system-level components are large, complex, and prohibitively expensive. A major impediment to the widespread commercialization of this technology is the lack of an economical, compact, broadband, and high-intensity THz source. We have investigated broadband THz to IR emissions from randomly patterned metallic nanostructures as a potential economical and efficient broadband source for spectroscopic applications.

Available laser-based methods for the generation of THz light include optical rectification in nonlinear crystals^4^,^5^,^6^, transient current in photoconductive antennas^7^, and THz field emission from laser-induced air plasmas^8^,^9^,^10^,^11^,^12^. However, these techniques suffer from a narrow emission bandwidth and/or low conversion efficiency. Thus, using THz radiation for spectroscopic measurements and research in biomolecular dynamics is difficult because of the long acquisition times required. A considerable amount of research has been conducted on the use of femtosecond lasers to generate THz radiation from nanostructured metal surfaces, including planar (percolated) metal films^13^,^14^,^15^,^16^, corrugated films (shallow gratings)^17^,^18^,^19^,^20^, and ordered arrays of metallic nanoparticles^21^,^22^,^23^. However, the THz emission from the aforementioned sources is too weak for many applications.

In contrast to these previous studies, a randomly ordered metallic nanostructured surface was used in the present study. A metal film was deposited on a substrate with randomly arranged nanoscale pores. The metallic surface therefore acquired a nanoscale and predominantly sub-wavelength surface roughness, which substantially enhanced the light coupling in the films. This enhancement resulted from several absorption mechanisms. First, the low density of the surface structures can cause an antireflection effect since the random sub-wavelength surface textures form a graded refractive index at the air/solid interface^24^. Second, nanometer-sized surface structures can give rise to enhanced absorptivity from plasmonic effects^25^,^26^,^27^. The most common approach to couple photons and surface plasmons is to form a grating on the surface. Efficient coupling depends on matching the momenta of the photons and plasmons, which can be achieved whenever the difference between the photon and surface plasmon wave vectors is an integral multiple of 2*π*/Λ, where Λ is the grating constant. In the present study, we used a randomly roughened surface to realize momentum matching over a wide range of wave vectors^28^,^29^. In addition to providing the coupling mechanism, the surface roughness features also served to localize and further enhance the surface plasmons^30^.

After irradiating a surface with a femtosecond laser pulse, a fraction of the absorbed laser energy is retained in the surface layer of the sample and then dissipates into the bulk sample via heat conduction as residual thermal energy^31^,^32^. It has been determined that a significant amount of residual energy can be deposited in samples through surface roughness effects, exothermic chemical processes and ambient gas pressure effects^33^. The residual energy causes the temperature of the bulk sample to increase. After reaching thermal equilibrium, the bulk sample then acts as a thermal radiation source.

In this study, we identified strong THz to IR thermal radiation from a randomly roughened metallic surface. We optimized the design of the metal deposition parameters to realize mW-level radiation intensity and an ultra-broadband (~150 THz) frequency spectrum. An intense coherent THz signal was generated through the optical rectification process simultaneously with the strong thermal signal. The coherent THz signal was also enhanced with the pump fluence because the laser absorptivity was enhanced by the thermal effect of the metal surface.

## Results

### Experimental setup and sample characteristics

**Figure 1:**
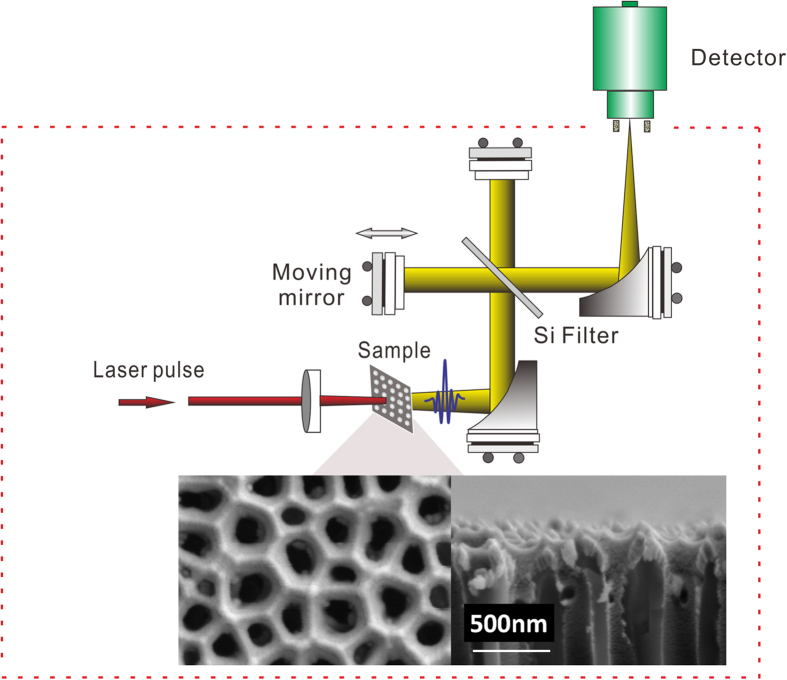
Schematic of the Fourier-transform Michelson interferometer. The magnified frame at the bottom shows scanning electron microscope (SEM) images of a top view (left) and of a cross-section view (right) of the metal film.

We used a Fourier-transform Michelson interferometer to quantitatively characterize the radiation spectra emitted by our samples. Figure 1 presents a schematic of the experimental setup. An amplified Ti:sapphire laser having a central wavelength of 800 nm, pulse energy of 1.3 mJ, pulse duration of 100 fs, and a repetition rate of 1 kHz was used. The metallic nanostructured sample was placed in the beam path with the metallic film surface facing the incident beam. The emission was collected from the side of the sample that faced away from the beam. The optical beam was focused normally on the sample with a spot diameter of 6 mm. A 0.4-mm-thick, high-resistivity silicon wafer was used as the THz to IR beam splitter. The radiation was detected using a Golay cell that was equipped with a 6-mm-diameter diamond input window (Microtech SN:220712-D), which exhibited a nearly flat response over a broad spectral range (0.1–150 THz). The voltage signal from the detector was fed to a lock-in amplifier referenced to a 15 Hz modulation frequency. The system was purged using dry nitrogen gas to prevent absorption from water vapor in the ambient air.

The sample substrate was a 60-μm-thick anodic-aluminum-oxide (AAO) membrane with a 200 nm pore diameter (Whatman, Germany). The pore density was approximately 50%. The metal was deposited on the membrane by magnetron sputtering. The metallic thin films had a nominal thickness of 100 nm and exhibited a random nanoscale surface roughness because of the through-pore structure of the AAO membranes. Three different types of metals, ruthenium (Ru), platinum (Pt), and gold (Au), were used in the experiments. The magnified frame at the bottom of Fig. 1 shows scanning electron microscope (SEM) images of a top view (left) and of a cross-sectional view (right) of the 100-nm-thick Ru metal film. The surface topography of the film was similar for all of the tested metals. The representative images show a variety of surface structures, including voids and nanoprotrusions, on the metal film.

### Frequency spectra of thermal radiation

**Figure 2:**
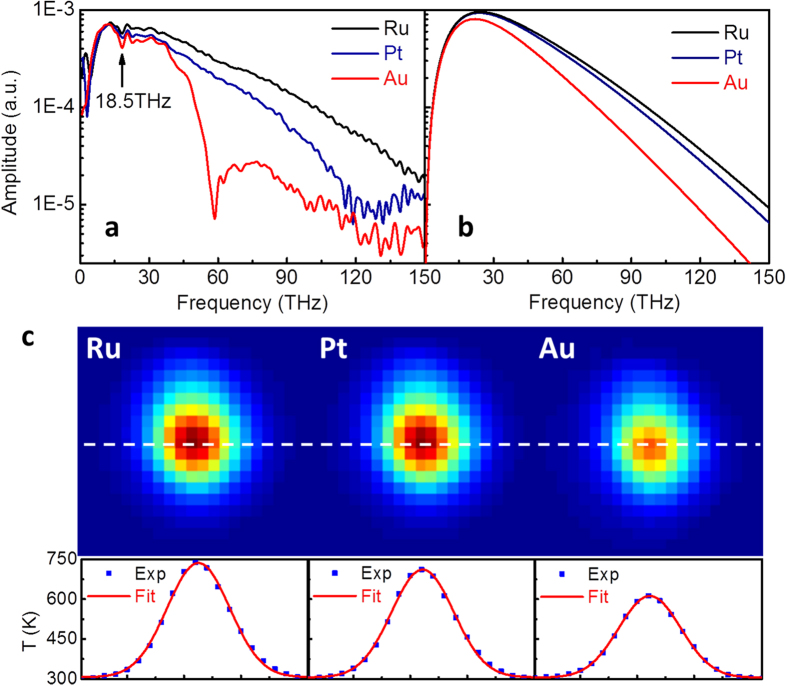
Frequency spectra and surface temperature measurements. (**a**) Measured radiation spectra for 100-nm-thick Ru, Pt and Au films; (**b**) simulated frequency spectra; (**c**) measured laser-induced surface temperatures of the samples; the bottom panel shows a cross section (white line) of the temperature distribution; blue squares denote the experimental data; red curves are Gaussian fits.

Figure 2a presents the radiation spectra for the 100-nm-thick nanostructured Ru, Pt and Au films that were measured using the experimental setup shown in Fig. 1. The signals for the three emitters were measured at identical pump fluence of 3.5 *mJ*/*cm*^2^. The signal bandwidths were ultra-broad, extending up to 150 THz for the Ru film (black curve). The spectral feature at approximately 18.5 THz resulted from the two-phonon absorption of the silicon wafer.

Figure 2b shows the frequency spectra that were simulated using Planck’s law for thermal radiation. The simulation temperature was measured using an IR thermal camera (Fig. 2c). The laser-induced surface temperature was sensitive to the intensity of the incident laser beam. Therefore, the measured surface temperatures followed a Gaussian distribution. The measured surface temperatures (Ru>Pt>Au) were consistent with our estimates for these values (see Supplementary Note 1).

The good quality of the fit demonstrates that our samples indeed behaved like grey bodies with a constant emissivity. The measured spectra are slightly different from the simulated spectra because of uncertainties in the system response, such as imperfect wave collimation, and the spectroscopic absorption of residual water vapor, the silicon wafer, and the substrate. Note that a dip appears at 60 THz in the frequency spectrum of the Au film. We have not been able to identify the origin of this dip.

For comparison, we also tested 100-nm-thick Ru, Pt and Au thin films deposited on polished sapphire crystal substrates with magnetron sputtering. These films were homogeneous and evenly distributed on the substrate. We are not able to provide measurements of radiation spectra, since the generated power is below the sensitivity of our calibrated detector.

### Power dependence and angular distribution

**Figure 3:**
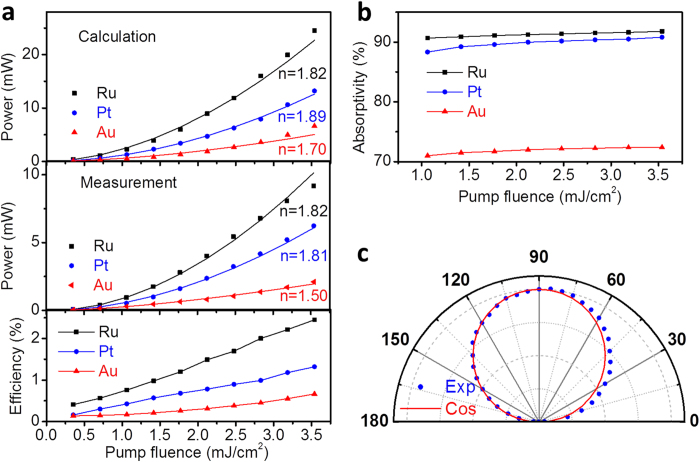
Power dependence and angular distribution. (**a**) Top: total radiation power calculated using the Stefan-Boltzmann law for Ru (black squares), Pt (blue dots) and Au (red triangles) films. The lines are the polynomial fitted curves; Middle: radiation power measured using a calibrated Golay cell; Bottom: calculated pump to THz to IR energy conversion efficiency; (**b**) measured absorptivity of Ru (black squares), Pt (blue dots) and Au (red triangles) films under different pump fluences; (**c**) angular distribution of THz to IR radiation; experimental data (blue dots) were fit to a cosine function (red curve).

The total thermal radiation power can be estimated using the Stefan–Boltzmann law *P* = *εσST*^4^, where *σ* = 5.67 × 10^−8^ *Wm*^−2^K^−4^ is the Stefan-Boltzmann constant, *ε* is the emissivity, *S* is the surface area, and *T* is the surface temperature. The surface temperature was measured under different pump fluences for the calculation. The calculated radiation power versus pump fluence is shown in Fig. 3a (top). The results for the Ru, Pt and Au samples could be fit using power laws with exponents of 1.82, 1.89 and 1.70, respectively. Figure 3a (middle) shows the dependence of the THz to IR radiation power on the pump fluence, as measured using a calibrated Golay cell. The exponents of the power laws for the Ru, Pt and Au samples were clearly consistent with the calculated values. We attributed the decrease in the measured exponent of the Au sample from 1.70 to 1.50 to the spectral feature at approximately 60 THz. Figure 3a (bottom) shows that the energy conversion efficiency of pump optical laser to THz to IR radiation linearly increased with the pump fluence. The maximum pump to THz to IR energy conversion efficiency for the Ru film reached 2.45%, which resulted from a maximum emitted radiation power of 24.5 mW. Further up-scaling of the radiation power is only limited by the available pump laser energy. Supposing we exploit the full sample area (25.4 mm in diameter) with a 3.5 *mJ*/*cm*^2^ laser fluence, the output power of the radiation will be up to the W-level.

The emissivity values in the power calculation were extracted using the measured absorptivity of each metal film, as shown in Fig. 3b. According to the Drude model, the appreciable enhancement in the absorptivity with the pump fluence resulted from an increased frequency of collisions between free electrons and thermally vibrating lattice atoms.

Notably, the measured power shown in Fig. 3a (middle) does not account for the spatial clipping by the Golay cell window. Figure 3c shows that the angular distribution of the THz to IR radiation was a circle that was tangent to the surface. The blue dots represent the normalized radiation power and are in excellent agreement with a cosine fit (red curve), as expected from Lambert’s cosine law. Therefore, a large portion of the emitted radiation was lost because of the size of the aperture of the Golay cell window and the unavoidable distance between the detector sensor and the sample surface.

### Spectroscopic measurement

**Figure 4:**
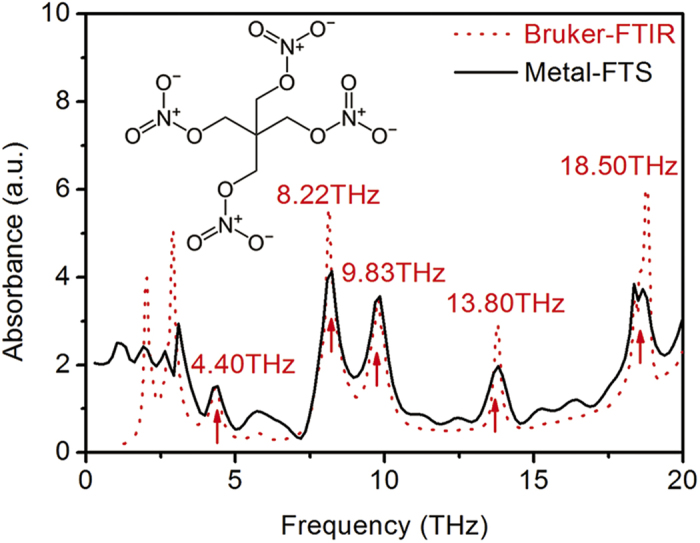
Spectroscopic measurement. Comparison of absorption spectra of PETN measured using our Fourier-transform Michelson interferometer (black solid line) and a commercial Bruker FTIR (red dashed line).

We demonstrated the capability of our Fourier-transform Michelson interferometer to make broadband spectroscopic measurements by measuring the absorption spectrum of a pentaerythritol tetranitrate (PETN) pellet sample using the setup in Fig. 1b. The results were compared to those obtained using a Bruker FTIR. The transmission spectroscopic results that were obtained using the two systems were in good agreement with each other (see Fig. 4).

### Polarization

**Figure 5:**
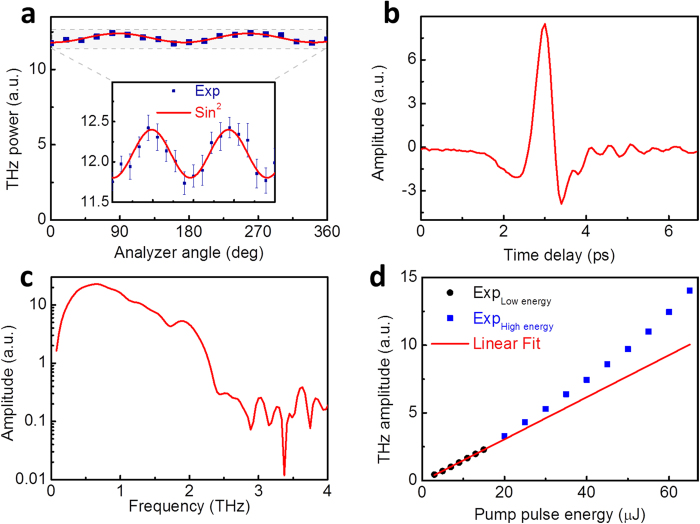
Polarization and coherent detection. (**a**) Measured energy of transmission as a function of the analyzer angle; inset shows an enlarged view of the THz polarization in the shaded area of the main panel; experimental data (blue squares) were fitted by a squared sinusoidal curve (red line); (**b**) time-domain waveform of THz signal as detected by EO sampling; (**c**) corresponding frequency spectrum; (**d**) peak-to peak amplitude of THz signals versus pump pulse energy; experimental data for pump energies below 15 μJ (blue dots) were fitted by a linear curve (red line); data for pump energies above 15 μJ (blue squares) deviated from the fitted line because of enhanced optical absorptivity.

It is well known that thermal radiation is unpolarized. We determined the polarization of the THz to IR radiation to completely characterize the beam. Representative results were obtained using the 100-nm-thick Ru sample. The polarization was determined by recording the THz power transmitted through a THz wire-grid polarizer positioned at different angles. A quartz filter with a transmission cutoff of less than 3 THz was used to match the effective bandwidth of the THz polarizer. Figure 5a shows the measured power as a function of the analyzer angle. Approximately 6% of the collected THz power could be accounted for using a squared sinusoidal fit, as expected for a linearly polarized beam.

### Coherent detection

We demonstrated the coherence of the linearly polarized THz wave by using a polarization-sensitive electro-optic sampling method to detect the THz signal. Figure 5b shows the time-domain waveform during a 6 ps interval for a pump energy of 60 μJ. Figure 5c shows the corresponding frequency spectrum with a bandwidth up to 3 THz. The THz signal was obtained at normal incidence. The optical rectification process that produced the observed coherent THz emission was induced by the broken symmetry of the second-order nonlinear current distribution in the non-centrosymmetric surface structure within the area irradiated by the laser beam.

Figure 5d presents a plot of the peak-to-peak amplitude of the THz electric field versus the pump pulse energy. Thermal effects on the coherent THz emission were negligible for pump energies below 15 μJ because the average surface temperature was less than 300 K. In this regime, the emitted THz electric field scaled linearly with the pump energy, thus corroborating the optical rectification process. Increasing the pump energy increased the surface temperature, and consequently, for pump energies above 15 μJ, the measured THz amplitude clearly deviated from the fitted linear curve. We attribute the observed super-linear law for the optical to THz conversion efficiency above a pump pulse energy of 15 μJ to an increase of optical absorptivity, and therefore of the laser field participating in the generation process. The minimum pump pulse energy for a detectable THz signal is as low as 1.5 μJ, which corresponds to a peak power intensity of 0.2 *GW*/*cm*^2^. This intensity can be achieved by focusing pulses from a femtosecond oscillator.

## Discussion

The overall generation efficiency in our study could be optimized further. According to the Stefan-Boltzmann law, the thermal radiation power is proportional to the temperature to the fourth power. Because the thermal radiation power depends on the thermal parameters of the metal, the type of metal should be selected to maximize the temperature. The absorptivity *A* is another important factor in determining the magnitude of THz to IR generation and consists of two components^34^:
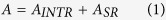
where *A*_*INTR*_ is the intrinsic absorptivity and *A*_*SR*_ is the corresponding contribution from the surface roughness. *A*_*SR*_ can be significantly enhanced by structurally modifying the surface. In our case, the surface structure could be easily modified by changing the pore diameter and density, depositing a suitable metal film thickness, or annealing after magnetron sputtering. This surface optimization should maximize the absorptivity.

Moreover, the rich variety of nano-scale sizes and shapes of the surface structure facilitate high absorptivity over a broad range of wavelengths^35^. The enhanced broadband absorption of the incident light increases the available laser options. We have verified that the amplifier can be replaced by an oscillator to produce comparable radiation power. The use of a compact laser can make the sample a good candidate for a THz to IR source for a variety of applications, such as a Fourier-transform spectrometer. Compared to commercial FTIR sources, such as mercury lamps (FIR: 0.15–20 THz) and globars (MIR: 10.5–200 THz), our sample can cover the entire THz to MIR frequency range (0.15–150 THz) in a single measurement. The intensity and bandwidth of the radiation can be controlled through setting appropriate pump fluences. Our sample can be used at room temperature, whereas mercury lamps and globars both require water cooling.

Our study shows that coherent and incoherent THz radiation is generated simultaneously from our metallic nanostructured sample. This unique feature opens up new avenues in biomedical research. The biological effects induced by exposure to high-power incoherent THz radiation that have been reported in previous studies^36^ could be probed using this coherent THz technique *in situ*.

In conclusion, we have demonstrated that a random metallic nanostructure can be used to up-scale the THz power of compact, laser-driven tabletop systems, representing a new platform for exploring broadband THz emission from artificial nanostructured materials.

## Methods

### Frequency spectrum simulation

Figure 2b shows the simulated radiation spectra, which are fitted to the formula for a grey body (using Planck’s law modified by an emissivity *ε*)
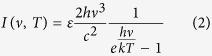
where *I*(*V*,T) is the power radiated per unit area of the emitting surface per unit solid angle per unit frequency, *h* is Planck’s constant, *c* is the speed of light in vacuum, *k* is Boltzmann’s constant, *v* is the frequency of the electromagnetic radiation, and *T* is the absolute temperature of the body. After calibration with a black-body source with a known temperature, the emissivities of Ru (*ε*_*RU*_ = 91.8%), Pt (*ε*_*PT*_ = 90.8%) and Au (*ε*_*AU*_ = 72.4%) were extracted. These values were in reasonable agreement with the measured absorptivities, as shown in Fig. 3b. We used the Gaussian distribution of the surface temperature to calculate the total radiation spectrum by integrating over the entire irradiated area.

### Electro-optic sampling method

In our experiments, we used an amplified Ti:sapphire laser having a central wavelength of 800 nm, pulse energy of 1.3 mJ, pulse duration of 100 fs, and a repetition rate of 1 kHz. The major portion of the laser was used as the pump beam, and the remaining portion of the laser was used as the probe beam. The horizontally polarized pump beam was weakly focused onto the sample surface to a spot size of approximately 6 mm at normal incidence. The THz radiation generated from the sample was collected using off-axis parabolic mirrors and focused onto a 3-mm-thick zinc telluride (ZnTe (110)) detection crystal. The synchronized, co-propagating sampling pulse was also focused onto the detection crystal. The THz electric field induced an elliptical polarization in the probe beam proportional to the value of the instantaneous THz electric field. The ellipticity of the beam was measured using a differential detection configuration consisting of a quarter wave plate, a Wollaston prism, and differential diodes.

## Additional Information

**How to cite this article**: Zhang, L. *et al.* High-power THz to IR emission by femtosecond laser irradiation of random 2D metallic nanostructures. *Sci. Rep.* **5**, 12536; doi: 10.1038/srep12536 (2015).

## Supplementary Material

Supplementary Information
