# Supplementary material for: High-power THz to IR emission by femtosecond laser irradiation of random 2D metallic nanostructures
Source: Sci Rep. 2015 Jul 24;5:12536. doi: 10.1038/srep12536 (PMC4513341; doi:10.1038/srep12536)
Supplement: Supplementary Information [file srep12536-s1.docx]

**High-power THz to IR emission by femtosecond laser irradiation of random 2D metallic nanostructures**

Liangliang Zhang^1,†^, Kaijun Mu^1^, Yunsong Zhou^1^, Hai Wang^1^, Cunlin Zhang^1^ & X.-C. Zhang^2^

^1^ Department of Physics, Capital Normal University, No.105 XiSanHuan BeiLu, Beijing 100048, China

^2^ The Institute of Optics, University of Rochester, Rochester, New York 14627, USA

**Supplementary Note 1. Comparison of the surface temperatures.**

Direct absorption of laser energy is a factor that influences the surface temperature. For our nanostructured metallic surfaces, the absorptivity was extremely enhanced. The absorptivity values for three different metal films with identical structured substrates (and therefore identical surface structures) were measured. The surface temperature is proportional to $T_{s}\propto\frac{A\sqrt{a}}{k}$ when a bulk metal reaches thermal equilibrium, where $A$ is the absorptivity of the incident laser, $a$ is the thermal diffusivity, and $k$ is the thermal conductivity^38,39^. Using the parameters of Ru, Pt and Au, it was calculated that$(\frac{A\sqrt{a}}{k})_{Ru}=0.0049$, $(\frac{A\sqrt{a}}{k})_{Pt}=0.0046$ and $(\frac{A\sqrt{a}}{k})_{Au}=0.0018$. Therefore, Ru metal film exhibits the highest temperature, the temperature of Pt film is in the middle, and the temperature of Au film is the lowest.

**Supplementary References**

1. Vorobyev, A., Kuzmichev, V., Kokody, N., Kohns, P., Dai, J. & Guo, C. Residual thermal effects in Al following single ns- and fs-laser pulse ablation. *Appl. Phys. A* **82**, 357-362 (2006).
2. Ready, J. *Effects of high-power laser radiation* (Academic Press, New York 1971).
